# Role of Alginate Composition on Copper Ion Uptake in the Presence of Histidine or Beta-Amyloid

**DOI:** 10.3390/molecules27238334

**Published:** 2022-11-29

**Authors:** Cynthia Regina Albrecht Mahl, Rogério Aparecido Bataglioli, Guilherme Bedeschi Calais, Thiago Bezerra Taketa, Marisa Masumi Beppu

**Affiliations:** School of Chemical Engineering, University of Campinas, UNICAMP, 500 Albert Einstein Av., Campinas 13083-852, SP, Brazil

**Keywords:** alginate, histidine, beta-amyloid, copper adsorption, Alzheimer’s disease

## Abstract

The anomalous interaction between metal ions and the peptide beta-amyloid is one of the hallmarks of Alzheimer’s disease. Metal-binding biopolymers, including polysaccharides, can elucidate the fundamental aspects of metal ions’ interactions with biological tissue and their interplay in Alzheimer’s disease. This work focuses on the role of the alginate composition on Cu(II) adsorption in the presence of histidine or β-amyloid, the peptide associated with the progression of Alzheimer’s disease. Alginate samples with different mannuronic/guluronic (M/G) ratios led to similar Cu(II) adsorption capacities, following the Langmuir isotherm and the pseudo-second-order adsorption kinetic models. Although the presence of histidine produced up to a 20% reduction in the copper adsorption capacity in guluronic-rich alginate samples (M/G~0.61), they presented stable bidentate chelation of the metallic ion. Chemical analyses (FTIR and XPS) demonstrated the role of hydroxyl and carboxyl groups in copper ion chelation, whereas both crystallinity and morphology analyses indicated the prevalence of histidine interaction with guluronic-rich alginate. Similar results were observed for Cu(II) adsorption in alginate beads in the presence of beta-amyloid and histidine, suggesting that the alginate/histidine system is a simple yet representative model to probe the application of biopolymers to metal ion uptake in the presence of biological competitors.

## 1. Introduction

Alzheimer’s disease (AD) is the main cause of dementia among the elderly, leading to a loss of memory and restriction of cognitive functions that severely affect the patient’s life due to the malfunctioning or death of nerve cells, which causes irreversible brain changes [1]. Alterations in the brain of AD patients include the presence of abnormal plaques formed by the deposition of fibrillar beta-amyloid (βA) around the neurons and tangles, attributed to the accumulation of the tau protein inside the neurons [2]. The prevalence of  βA structures led to the formation of the amyloid cascade hypothesis to explain the emergence of the degenerative neural process. This hypothesis links the beginning of AD to the abnormal processing of the amyloid precursor protein, which is connected to the production of the βA peptide and facilitates the formation of both plaques and tangles and then irreversible neuronal death [3,4,5]. Evidence that reinforces this hypothesis includes (i) the presence of βA as the main component of the neural plaques in patients with AD; (ii) the mutations found in patients with AD led to an increase in the production levels of βA; and (iii) the toxicological effects associated with higher levels of βA in in-vitro and in-vivo experiments reflect the alterations observed in AD [6,7].

Several studies report higher levels of copper ions associated with patients with AD [8,9,10]. Copper is a micronutrient in human metabolism that contributes to catalyzing a series of biological processes (e.g., respiration and cell growth). This element also interacts with enzymes in the nervous system, regulating various steps in brain neurons [11]. In excess, however, copper forms reactive oxygen species (ROS), such as H_2_O_2_, O_2_, and OH^-^ [12], increasing brain oxidative stress. In AD, copper ion coordination with the peptide  βA has also been linked to the formation of ROS, potentializing the toxicity of the amyloid plaques [8,9]. Streltsov and colleagues describe the preferential binding of copper ions with βA in a distorted six-coordinated arrangement involving three histidine residues (His-6, His-13, and His-14), the carboxylate side chain of Glu-11 or Asp-1, and one axial water molecule [13,14].

Since high levels of βA have been linked to neural-degenerative diseases, studies have focused on using chelating agents for metal binding and solubilization of the amyloid plaques [15,16]. For instance, Cherny et al. described the application of clioquinol, a Cu/Zn chelating agent that decreases brain βA deposits by 49% in vivo [15]. Although promising, the application of chelating agents must fulfill various requirements for their use in AD treatment, such as the ability to cross the blood–brain barrier, limited alterations in brain homeostasis, and high affinity of the targeted ion species [16,17].

Alginate enables a broad spectrum of functionalities, including the adsorption of heavy metals for clinical and environmental purposes. It is a linear polymer of high molecular weight formed by units of β-D-mannuronic (M) and α-L-guluronic (G) acid found in large proportions in brown algae, including *Macrocystis*, *Laminaria*, and *Ascophyllum* species [18]. The ratio (M/G) and sequence of these monomers (MM, MG, GG) vary based on the source, harvest location, and season [19,20] and impact polymer reactivity, mechanical, and rheological properties [21]. Like other biopolymers, alginate is biodegradable, biocompatible, and non-toxic, with relevant applications ranging from biomedical products to the food industry [22]. The biocompatible and pH-responsive properties of alginate-based materials also favor their use in pharmaceutical products, including drug delivery [23,24], wound healing [25], and implants [26]. The affinity of alginate to metallic ions, such as copper, also facilitates the application of the alginate-based system as an adsorbent for environmental purposes.

The adsorption capacity of alginate is highly affected by the proportion of M/G units and the conformation of alginate chains, forming a zigzag structure due to the alignment of G sequences that can accommodate divalent cations in the coordination sites between the alginate chains [27]. Alginate sequences rich in G blocks present higher selectivity to divalent cations attributed to the presence of bivalent coordination sites on each monomer, as opposed to single coordination with M blocks, which leads to weaker interaction with divalent cations [28]. The egg-box model illustrates the interaction of alginate and metal ions and considers the association of two or more alginate chains to form a structure connected by calcium ions in the polymer chain vacancies [29]. The formation of the strong metal-polymer complex of alginate with metal ions has also been extensively described, leading to a suitable product for wastewater treatment due to its high adsorption capacity and low cost compared to other polymeric adsorbents [30,31]. This highlights the potential application of alginate to chelate copper ions in order to destabilize βA plaques for AD treatment, given that the polymer properties are thoroughly understood.

Herein, we investigated the role of alginate composition (M/G ratio) on copper ion uptake in the presence of histidine or βA. By testing alginate as a competitor for metal adsorption with histidine, or the histidine-bearing βA peptide, this work provides insights into the metal-biopolymer interactions that can be tailored to mitigate the buildup of copper ions that occurs in biological systems during AD.

## 2. Results and Discussion

### 2.1. Adsorbent Characterization

The ionic crosslinking process led to the formation of alginate particles with diameters ca. 3 mm (Figure S1 and Table S1 in Supplementary), leading to more irregular-shaped spheres for AlgGel and no shape or size change after copper binding. This crosslinking process relies on the binding of divalent calcium ions to alginate G groups that form electronegative cavities capable of trapping the cations via ionic interaction, resulting in the crosslinking of the chains and thereby creating the structure called “egg box” [30]. The same process may occur on both AlgCol and AlgGel that, even though the M/G is different, both possess enough G groups and can withstand the crosslinking process to some extent.

**Figure 1:**
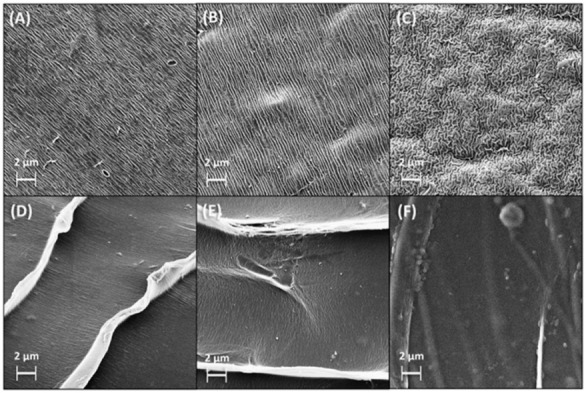
SEM images for lyophilized spheres of (**A**) AlgGel, (**B**) AlgGel + Cu(II), (**C**) (Cu(II) + His) + AlgGel, (**D**) AlgCol, (**E**) AlgCol + Cu(II), and (**F**) (Cu(II) + His) + AlgCol.

Scanning electron microscopy (SEM) revealed that AlgGel exhibited an irregular, grooved-surface structure, and AlgCol presented a more homogeneous surface, with the formation of layers with a wave-like structure, in which the groove structure is considerably less visible (Figure 1A–F). Compared to pristine alginates, copper adsorption did not cause substantial changes in surface morphology. The copper adsorption in the presence of histidine caused an increase in the adsorbent bead surface roughness and the disorientation of the grooved structure, possibly associated with the coupled interaction of both copper and histidine with the adsorbent surface.

**Figure 2:**
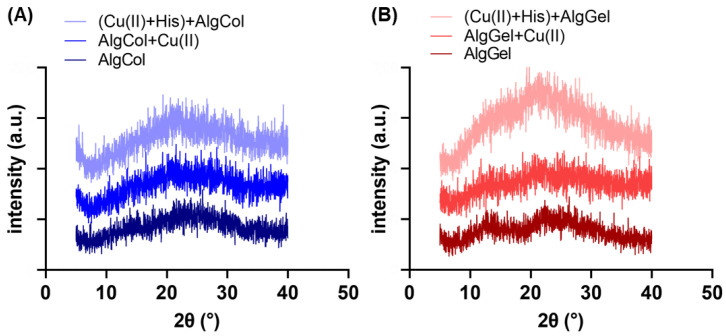
XRD spectra for (**A**) AlgCol and (**B**) AlgGel before and after copper adsorption.

The changes in the adsorbent crystallinity were assessed by *X*-ray diffraction (XRD) (Figure 2A,B). Both pristine alginates exhibit the characteristic halos 2ϴ~13.7° and 21° in the XRD spectra [32]. The alginate with the highest amount of guluronic groups, AlgGel, appears to have a structure slightly more crystalline than that of AlgCol, which has a high amount of M blocks that do not form the “egg-box” polymer structure, leading to a more spatially disordered gel structure for the later [33]. The adsorption of copper ions in the alginate led to broader peaks in the XRD for both alginates, especially at 2ϴ = 13.7°. This is possibly attributed to the metal chelation to alginate functional groups, which breaks the interchain hydrogen bonds and disrupts the polymer crystallinity. Gu et al. reported the same behavior in XRD spectra for copper ions adsorption on carboxymethyl-chitosan nanoparticles [34]. Furthermore, part of the calcium ions used to promote the crosslinking of the chains is exchanged by copper ions. As the copper ion is more prominent, a disturbance may occur at the polymer chain distribution of the already-formed sphere [35].

The presence of histidine significantly increased the peaks in the XRD spectra, particularly for AlgGel. Anuradha (2016) grew an L-histidine monohydrate monohydrochloride crystal by recrystallization technique, determining the material’s most intense XRD peak is found at a 2ϴ of c.a. 23.2° [36]. This suggests that the increase in the XRD peaks for AlgGel may reflect the attachment of histidine molecules in alginate structure, rather than a chain rearrangement and increase in polymer crystallinity in the presence of histidine. The reduced variation in XRD peaks for AlgCol after histidine contact, with a smooth halo at 13.7° and maintenance of the peak at 2ϴ = 21°, suggests little or no change in its crystallinity for AlgCol. The less noticeable changes after adding histidine to the system indicate that this amino acid interaction with alginate rich in M units is less extensive than the one rich in G units.

**Figure 3:**
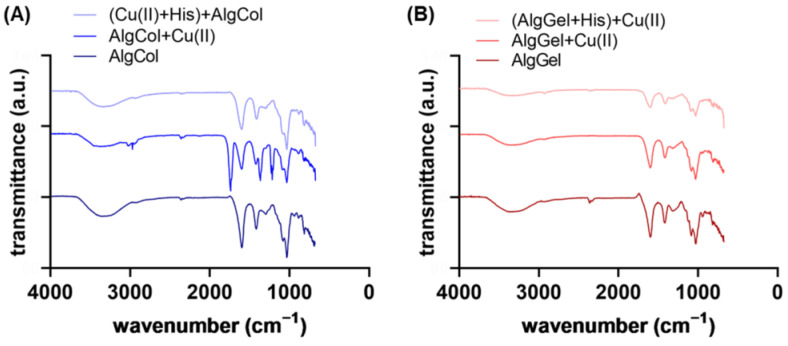
FTIR spectra for (**A**) AlgCol and (**B**) AlgGel before and after copper adsorption.

Infrared spectra evidenced features of alginates and the differences between AlgCol and AlgGel composition (Figure 3A,B). Characteristic bands for alginate are observed at 3340 cm^−1^, attributed to O-H hydrogen bonds stretching; 2930 cm^−1^ due to C-H stretching vibrations; 1600 cm^−1^, which corresponds to the COO^−^ asymmetric stretching of carboxylate anions; 1410 cm^−1^, attributed to vibrational deformation of the O-H bond with contribution from the carboxyl group C-O stretching; and at 1295 cm^−1^, 1080 cm^−1^, 1030 cm^−1^, and 940 cm^−1^, attributed to the stretching of the C-O and C-C bonds of uronic acid residues [37,38,39,40]. Those bands may also indicate the dimerization of poly-G-sequences with the calcium ions of the crosslinking agent on its interior [41]. The main changes from one alginate type to another are found in 1320, 1100, and 1080 cm^−1^ (characteristic bands of guluronic acid) and at 1290 cm^−1^ and 880 cm^−1^, attributed to mannuronic acid residues.

No significant difference was found in the spectra after adding histidine to the AlgCol system, even after 24 h of contact between AlgCol and histidine (Figure S3 and Figure 2). However, a slight intensity reduction in the overall spectra was observed for all the AlgGel peaks. As for the XRD analysis, this result may indicate a predilection of histidine interaction for the G-units-rich alginate. Contrarily, di Cocco and colleagues reported the interaction of alginate with four different amino acids after probing the protons coming from alginate M-units with the high-sensitive NMR technique [42]. This suggests that an interaction between alginate and histidine may occur. Still, confirming it with the Fourier-transformed infrared spectroscopy (FTIR) spectra was difficult because the interaction can also be affected by the contact position between the ATR crystal and the sample.

Copper ion adsorption led to both a slight decrease and a shift in the bands at 3340 cm^−1^ (O-H bonds) and at 1600 cm^−1^ and 1410 cm^−1^, which corresponds to the COO^−^ asymmetric and symmetric stretching. The peak at 1738 cm^−1^, observable in AlgCol + Cu(II), corresponds to the stretching vibrations of the C=O group of saturated aliphatic carboxylic acids, which is very strong when hydrogen is bonded to the carboxyl group or due to dimerization. The peak at 1216 cm^−1^, C-O str., confirms the presence of dimers in this sample. Sartori et al. (1997) reported that the peak at 1410 cm^−1^ is responsible for ionic bonds and that the replacement of sodium with calcium ions in the formation of alginate thin films causes the changes observed herein [43]. The ion exchange modifies the chemical environment surrounding the carbonyl group due to the changes in the cation size and charge density. In the present work, this band displacement may be associated with removing calcium ions and inserting copper ions in the biopolymer matrix [43,44]. However, the alterations of these peaks are subtle compared to the exchange of sodium by calcium (Figure S4 and Figure 3). The peak at 940 cm^−1^ disappeared after the copper ions’ adsorption, indicating the metal’s interaction with the uronic acids of the biopolymer. Bertagnolli (2013) also observed this peak’s disappearance after chromium adsorption on commercial alginate beads [45].

The Infrared spectra also provided information on the copper ion chelation in the alginate matrix. Mehrotra and Bohra (1983) suggested that the metal-carboxylate complex may have different types of coordination: ionic or uncoordinated, monodentate coordination, bidentate coordination via chelation, and bidentate coordination in a bridge [46]. The frequency of the COO^-^_asymmetric_ (1600 cm^−1^) and COO^-^_symmetric_ (1410 cm^−1^) bands is dependent on the structure of the carboxyl group, the nature of the solvent, the ligand, and the metal element. These bands can be used to probe the interaction between the alginate and the metal ions [47,48]. The interval among these bands, that is, $∆\nu ={\mathrm{COO}}_{asymmetric}^{-}-{\mathrm{COO}}_{symmetric}^{-}$, can indicate the structure of the carboxylate. According to Nakamoto (1978) and Deacon and Phillips (1980), values of $∆\nu_{complex}<∆\nu_{Na}$, suggest bidentate coordination via chelation; $∆\nu_{complex}≅∆\nu_{Na}$, indicates bridged bidentate coordination and $∆\nu_{complex}>∆\nu_{Na}$, represents monodentate coordination [49,50]. $∆\nu_{complex}$ stands for the wavenumber interval in the studied complex, while $∆\nu_{Na}$ represents the interval in the sodium alginate. Both alginates presented $∆\nu_{complex}<∆\nu_{Na}$, indicating bidentate coordination of copper ions via metallic chelation (see Table S2 in the SI).

The *X*-ray photoelectron spectroscopy (XPS) technique is a relevant tool for understanding the adsorption mechanism at the molecular level and complementing XRD and FTIR results. Even though XPS analyses alone do not directly indicate the overall composition of the alginate matrix, the results are useful for a first indication of the surface composition of the alginate beads. The assessment of the adsorbent AlgGel was chosen due to its stability in competitive adsorption experiments when compared to AlgCol (see Section 2.2). The presence of nitrogen in AlgGel (Table 1) can be explained by an inefficient extraction process that leaves behind traces of algae proteins [51,52]. In contrast, the signal for calcium is due to the crosslinking process of the alginate spheres promoted by calcium chloride. Copper presence was observed in the samples after adsorption, with a decrease in copper signal for the sample containing histidine, and this result is in agreement with the adsorption processes (see Section 2.2 for a more detailed explanation of the adsorption data and mechanisms). The XPS survey also indicates a reduction in the calcium concentration on the alginate surface with copper adsorption. This result may represent the ion-exchange mechanism between Ca^2+^ and Cu^2+^ during adsorption, corroborating the literature [28,30,53,54,55]. The crosslinking process with CaCl_2_ was conducted at pH 7, whereas the copper adsorption assays occurred at pH 5. The assessment of alginate transformations during pH alterations is intended to enable a better understanding of the chemical transformations the adsorbent undergoes and how it may affect the ion-exchanging processes (Na^+^ to Ca^2+^ and Ca^2+^ to Cu^2+^). The decrease in the storage solution pH (from 7 to 5) of AlgGel beads also led to a reduction in calcium concentration, and the absence of nitrogen reflected an increase in oxygen concentration. Considering the alginate pKa, the sphere may have released calcium ions at lower pH levels into the medium (H_2_O).

**Figure 4:**
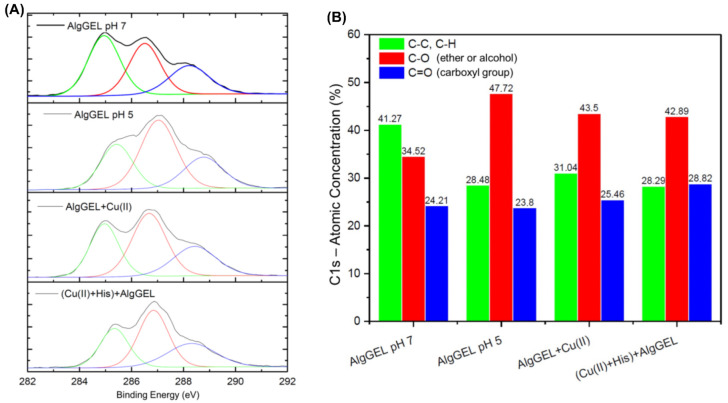
(**A**) High-resolution XPS spectra for carbon (C1s) and its respective deconvolutions for AlgGel before and after copper adsorption. The pH values represent the solution conditions for bead storage after crosslinking. (**B**) Deconvoluted peaks for C1s binding energies and their respective atomic concentration in each studied system.

Carbon binding characteristics were investigated by analyzing the high-resolution XPS spectra of C1s to probe how the copper binding and histidine interaction affect the AlgGel properties (Figure 4A). The Binding Energy (BE) peak of 285 eV is attributed to the C-C/C-H bonds; these groups are generally used for instrument energy calibration [56]. The other peaks can be attributed to the C-O bond of alcohols and ethers and the C=O bond of carboxyl groups with BE of 286 and 288 eV, respectively [56,57]. These organic functions are typical of algae polysaccharides, and the variations in the BE of the carbon atom are due to the different electron densities of the groups. Figure 4B identifies the peaks that underwent deconvolution based on the C 1s binding energies and presents the atomic concentration of each peak. The increase in the C-O signal under more acidic conditions indicates that lower pH values increase the exposure of functional groups on the surface of the AlgGel beads. In a more acidic solution, the functional groups are more exposed because fewer calcium ions are present to complex them, as part of them was released. The apparent increase in the concentration of the bonds corresponding to functional groups promotes the decrease in the signal of the C-C bonds. The copper ion adsorption increases the signal of the C=O bonds of the carboxyl functional groups, suggesting the participation of these groups in the capture of copper ions (Figure 4B). The same can be observed when histidine is added to the system, which indicates the formation of a carboxyl-metal complex in which oxygen atoms donate electrons to metal ions. In this way, the electron density of the carbon atom decreases, causing the BE to drop from 288.77 to 288.43 eV. The low-resolution spectra for copper are presented in Figure S2 in Supplementary, evidencing the predominance of Cu(II) species in the alginate beads based on the peaks that correspond to Cu 2p2/3, and Cu 2p1/2 [58,59,60,61,62].

### 2.2. Adsorption Kinetics

**Figure 5:**
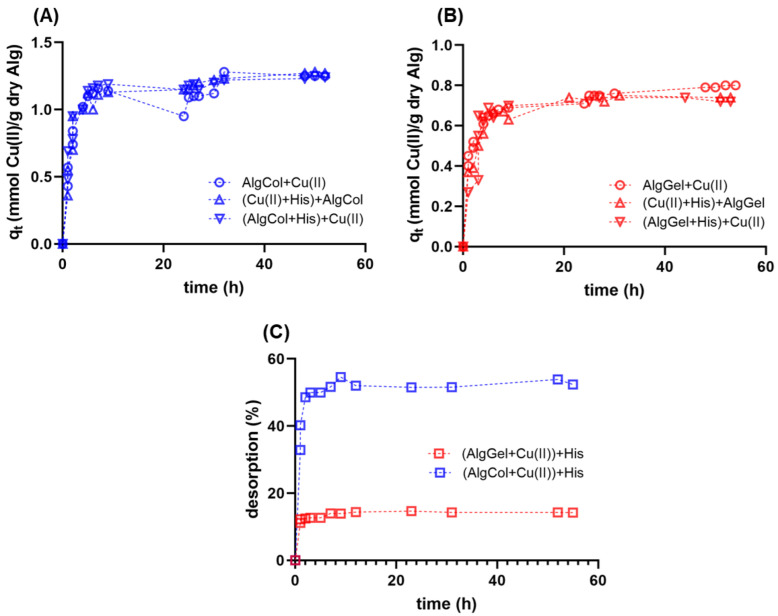
Adsorption kinetics for copper ions in alginate beads at 25 °C and pH 5.0 using (**A**) AlgCol and (**B**) AlgGel as adsorbents. Experiments were conducted without histidine (Alg + Cu(II)), with alginate beads treated with histidine before copper adsorption ((Alg + His) + Cu(II)), and with histidine mixed with copper before alginate beads addition ((Cu(II) + His) + Alg). The initial concentration of copper nitrate is 0.47 mmol L^−1^. (**C**) Desorption kinetics of copper ions from alginate beads after adding histidine at 25 °C and pH 5.0.

Adsorption kinetics experiments are essential for understanding the transport mechanism that governs metal ion adsorption for the design of efficient adsorbents. Herein, we investigated the adsorption of Cu(II) in alginate beads over time; both alginates tested presented a fast, two-step mechanism, reaching equilibrium after 25 h (Figure 5). Previous studies report the same behavior for metallic ion adsorption in alginate-based adsorbents with one rapid, minute-long initial adsorption, followed by a more cadenced adsorption step that lasts several minutes up to hours [63]. Kleinübing and colleagues also studied the adsorption of copper ions for an extracted alginate and the pristine algae, describing similar kinetic behavior but smaller equilibrium adsorption capacities, possibly due to the small particle diameter of the adsorbent [64].

In the present work, two approaches were tested to investigate how the presence of histidine impacts the kinetics and adsorption capacity of copper ions in alginate particles. First, alginate beads were treated with histidine before copper ions adsorption. In the second approach, histidine was added to the copper solution, followed by the later addition of alginate particles for metal uptake. Both cases did not lead to qualitative changes in the adsorption kinetics profile of copper ions compared to the case in the absence of histidine (Figure 5A,B). This result highlights the high affinity of alginate compared to histidine for copper ions uptake, regardless of the polysaccharide composition, reinforcing its use for copper ions uptake in the presence of histidine-bearing proteins.

The adsorbent performance can be better assessed by determining the parameters in the adsorption processing, including the adsorption rate and the maximum amount of the adsorbate bound. These parameters can be obtained by fitting the adsorption results over time to kinetics models. By assessing how well the adsorption data fit a specific model, one may also find the steps that limit the adsorption process, such as the solute transport through the fluid phase, the intraparticle diffusion, or the chemical reaction involved in the adsorption process. The mechanism that limits the process designates it as physical adsorption—i.e., controlled by the mass transfer resistance—or chemisorption, determined by the chemical reaction [65]. The pseudo-first-order model is used to represent the physical adsorption and is based on the following equation (Equation (1)):

(1) $$q_{t}=q_{e}\left(1-e^{-k_{1}t}\right)$$

$q_{t}$ and $q_{e}$ are the adsorption capacity (mmol adsorbate/g adsorbent) at the time t (h) and equilibrium, respectively, and $k_{1}$ is the kinetic constant (h^−1^) for the pseudo-first-order model [66]. The pseudo-second-order model considers that the adsorption follows a second-order chemisorption process, described by the following equation (Equation (2)):

(2) $$q_{t}=\frac{q_{e}^{2}k_{2}t}{1+q_{e}k_{2}t}$$

$q_{t}$ and $q_{e}$ represent the adsorption capacity (mmol adsorbate/g adsorbent) at the time t (h) and equilibrium, respectively, and $k_{2}$ is the pseudo-second-order model’s kinetic constant (h^−1/2^) [67].

Adsorption kinetics data were fitted to both models to investigate the impact of the alginate composition on the adsorption mechanism and compare the kinetic adsorption parameters for each case (Table 2). The pseudo-second-order model presented a better fit for both alginates based on the *R*^2^ and the χ^2^ values. This result indicates that the copper ion uptake is a chemisorption process, i.e., the adsorption rate is limited by the reaction between the metal ions and the active binding sites in the alginate particles [68], which possibly takes place via covalent bonds formed between the copper ions and the carboxylate groups of alginate chains.

The parameters retrieved from the pseudo-second-order model indicated higher $k_{2}$ values for AlgGel and higher $q_{e}$ for AlgCol samples in all adsorption conditions investigated. Zheng et al. reported the same q_e_ trend for alginate with various compositions [69]. The increase in the adsorption capacity with the reduction in the M/G ratio was attributed to the change in the ion-exchanging mechanism that governs the adsorption of copper ions [70]. Zheng et al. described that the divalent cations, such as Ca^2+^, bind cooperatively to G-blocks, forming cavities that facilitate metal binding sites and increase gel stability. Because multiple polymer chains chelate them, calcium ions bound to G-blocks are challenging to replace by other metal ions during adsorption. However, calcium ions chelated by MG blocks are not bound to different polymer chains. They are more prone to ion exchange with copper, leading to higher adsorption capacity values for the alginates with lower GG fractions [69]. Notably, the differences in alginate adsorption capacities correspond to the kinetics tests with an initial concentration of copper ions (0.47 mmol L^−1^) lower than the plateau concentration values in the equilibrium during isotherm experiments (see next section), meaning the differences in $q_{e}$ correspond to adsorption experiments under more diluted conditions. Despite the higher porosity of AlgGel compared to AlgCol beads (see SEM results in the previous section), it remains challenging to relate this to the higher $k_{2}$ values of AlgGel, considering the chemisorption nature of the metal ion uptake by alginate particles in this study.

The presence of histidine in the reaction medium caused a reduction in $k_{2}$ in nearly all adsorption systems tested and no significant change in the $q_{e}$, compared to the adsorption experiments without histidine. The decrease in the adsorption kinetics may be attributed to the interaction between the histidine and the copper ions, which may impair the migration and binding of the metal ions onto the alginate particles. The maintenance of the $q_{e}$ values in the presence of histidine reinforces the strong affinity of alginate for copper ions. Taketa and colleagues investigated the adsorption of copper ions in chitosan beads in the presence of histidine [71]. The authors reported a reduction in $k_{2}$ and $q_{e}$ in the presence of the amino acid, illustrating a competition for copper ions between this amino acid and the chitosan beads. The authors also reported lower values of both $k_{2}$ and $q_{e}$ for pristine chitosan compared to those obtained for alginate samples. These findings reinforce the stronger affinity of alginate particles and copper ions, which may be of interest for applications that involve competition for metal ions with proteins bearing histidine residues.

This study also investigated the addition of histidine after the adsorption of copper ions in alginate particles reached equilibrium (Figure 5C). AlgCol samples presented cumulative desorption close to 60% after histidine mixing, whereas the AlgGel sample led to nearly 15% desorption. This result suggests that the alginate composition substantially impacts the affinity of copper ions to alginate in the presence of histidine. The reduced desorption for AlgGel may be associated with an irreversible interaction of copper ions with G-group-rich alginates that form binding pockets that strongly chelate metal ions in a bidentate complex [28]. The weaker interaction of copper ions with AlgCol, due to its higher number of M-groups that form a monodentate complex with the metal [28], may explain the higher desorption values observed for AlgCol. Despite the slight copper ions desorption with the later addition of histidine in AlgGel systems, these findings suggest that alginate species with high levels of GG blocks are slightly affected by histidine, or macromolecules bearing histidine residues during copper ion binding; this may be of interest for further investigation of alternatives for the capture of metal ions and destabilization of βA for AD treatment.

### 2.3. Adsorption Isotherms

**Figure 6:**
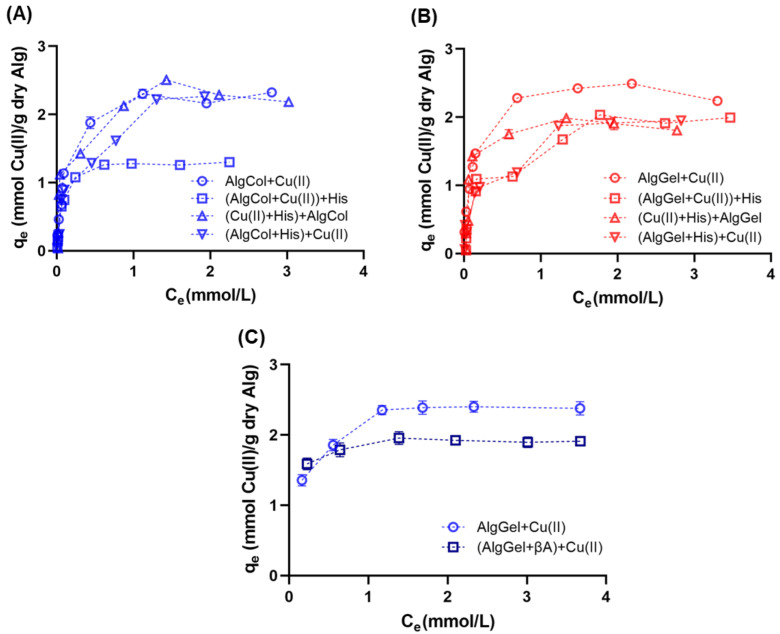
Adsorption isotherms of copper ions in alginate beads at 25 °C and pH 5.0 using (**A**) AlgCol and (**B**) AlgGel as adsorbents. Experiments were conducted without histidine (Alg + Cu(II)), with alginate beads treated with histidine before copper adsorption ((Alg+His) + Cu(II)), with histidine mixed with copper before alginate beads addition ((Cu(II) + His) + Alg), and with histidine added after copper ion adsorption ((Alg + Cu(II)) + His). (**C**) Adsorption isotherms of copper ions in AlgGel beads at 36.5 °C and pH 5.0 with and without the addition of βA to copper ions before the AlgGel mixture.

Analysis of the adsorption isotherm is critical for investigating adsorbent performance, as well as understanding adsorbent/adsorbate interactions at specific processing conditions. Adsorption isotherms indicated similar levels of copper ion adsorption for both alginates in the equilibrium, suggesting that the M/G ratio does not affect the copper ion uptake in alginate (Figure 6A,B), which Kleinübing and colleagues also observed for copper ion adsorption in alginate with different compositions. The reduced impact of the M and G monomers in metal ion adsorption may reflect the strong affinity of certain metal ion species for alginate binding sites that adsorb on the functional groups of both monomers, as observed for Cu(II) and Pb(II) adsorption [64,72]. In contrast, metal species with limited adsorption capacity, such as Cd(II) [30], reinforce the preference for one or another group in alginate, evidencing the impact of the M/G ratio on the metal uptake.

The presence of histidine impacted the adsorption profile of copper ions in alginate beads in most of the scenarios investigated. AlgCol beads presented a substantial drop in their adsorption capacity for the later addition of histidine to the reaction media ((AlgCol + Cu(II)) + His). In contrast, histidine addition before or during alginate beads addition does not affect the adsorption. For the system in which histidine was added later, a reduction in the adsorption capacity of AlgGel beads occurred to a minor extent as compared to AlgCol. The substantial drop in the adsorption capacity of AlgCol may be attributed to the desorption of copper ions in the presence of stronger chelating agents, such as histidine, due to the prevalence of MM blocks that form weaker interactions with copper ions, in contrast to the bidentate chelation of copper ions in the GG groups more prevalent in AlgGel. This property illustrates the higher stability feature of this alginate sample as a competitor agent for copper ions adsorption.

Isotherm results fit two mathematical models that better assess the performance of these alginate adsorbents in the presence of histidine as an adsorbent competitor. The adsorption models describe the interplay between the adsorption concentration in the solid and fluid phases during equilibrium. Their parameters are determined by inputting the experimental data obtained under constant temperature. Traditionally used to describe the adsorption of gases, the Langmuir model was further adapted to describe the adsorption of solutes in the liquid phase, based on the hypotheses that (i) all the adsorption sites are energetically equivalent and (ii) the adsorbed species does not affect the adsorption in adjacent binding sites. The following equation expresses this model:

(3) $$q_{e}=\frac{bq_{max}C_{e}}{1+bC_{e}}\;$$

where $C_{e}$ is the adsorbate concentration in the liquid phase and $q_{e}$ is the adsorbent concentration in the solid phase, both at equilibrium; $q_{max}$ is the maximum adsorption capacity parameter; and b is the adsorption bonding constant [73]. The empirical, non-linear Freundlich model is also widely used to represent the adsorption process. This model assumes solute adsorption from a liquid phase in the solid heterogeneous surface, with different adsorption energies in each binding site. The following equation represents this model:

(4) $$q_{e}=K_{F}C_{e}^{1/n}$$

where $C_{e}$ is the adsorbate concentration in the liquid phase and $q_{e}$ is the adsorbent concentration in the solid phase, both at equilibrium; $K_{F}$ reflects the maximum adsorption capacity; and n is the constant that represents the heterogeneous surface [74].

All the adsorption systems investigated presented a better fit for the Langmuir model (Table 3) based on the *χ*^2^ and the R^2^ parameters, which assumes an energetically homogeneous surface with an adsorbate monolayer [75]. Both alginates tested presented similar $q_{max}$ for both alginates in the absence of histidine. These values are higher than those reported in the literature for copper ion adsorption, including the complexed chitosan-alginate beads (Table 3), demonstrating the relevant performance of both adsorbents for metal ion uptake. Results for pristine alginate also presented a slightly higher value for the interaction parameter *b* for AlgGel than AlgCol. This parameter represents the partition coefficient for the solute between the solid and the fluid phase, i.e., its value is proportional to the interaction between the adsorbent and the solute. Because copper ions bind strongly to GG blocks via bidentate chelation, compared to monodentate chelation by MM blocks [76], higher *b* values are expected for AlgGel based on the higher M/G ratio, compared to AlgCol composition. The values of *b* for both alginates are also higher than those obtained for copper binding in chitosan beads, as described by Taketa and colleagues [71], illustrating the stronger affinity of copper ions for alginate.

Compared to pristine alginate, the presence of histidine as an adsorbent competitor led to a 20% decrease in the $q_{max}$ values for AlgGel in all scenarios investigated. The AlgCol system presented a slight reduction in the $q_{max}$ values in the presence of histidine, with the most substantial reduction for the amino acid addition occurring after copper binding to alginate (close to 47% in $q_{max}$), reflecting the competition with histidine for copper ion uptake. The increase in the interaction parameter, *b*, for alginate in the presence of histidine for some of the conditions investigated may reflect the Langmuir model’s assumption that only considers single-component adsorbents, which suggests that the contribution of histidine to the adsorption process is also represented in the *b* parameter. Vieira and colleagues observed similar behavior for chitosan beads after crosslinking with glutaraldehyde for copper ion adsorption [77].

Copper ion adsorption in the presence of βA was tested only with AlgGel particles due to its better performance for copper binding in the presence of histidine. The literature reports the use of alginate oligomers to neutralize both the oxidating and inflammatory impacts caused by βA [78,79], despite lacking information on the effects of alginate copper ions uptake and further destabilization of βA. This experiment was conducted close to body temperature, but at pH 5.0, due to the precipitation of copper nitrate solution for preliminary investigations at pH 7.4 (data not shown). Compared to adsorption results at 25 °C, no changes in copper ion adsorption profile for pristine AlgGel were observed at 36.5 °C (Figure 6B,C). The presence of βA causes a drop of ~20% in copper ion adsorption, close to the results observed using histidine as the competing agent. Taketa and colleagues also observed the same trend using chitosan beads [71], confirming that histidine may be a cheaper yet still valid adsorbent competitor for screening biopolymer adsorbents in the presence of βA.

Isotherm model parameters also evidenced the reduction in the maximum adsorption capacity for the adsorption of copper ions in the presence of βA (Table 3). The increase in the affinity parameter for the Langmuir model for copper adsorption in the presence of histidine may also reflect some degree of interaction between the copper ions and the peptide, whose interaction cannot be decoupled from the alginate-Cu(II) interaction because the Langmuir model assumes single-component adsorbents, as described above for histidine. Overall, despite the slight drop in $q_{max}$, AlgGel is suitable for copper ions adsorption in the presence of βA and presents enormous potential for further investigation related to mitigating AD, ameliorating the inflammation caused by βA, and chelating metal ions for destabilization of βA aggregates. Future investigations may focus on understanding how the presence of alginate in the system affects the stability of βA particles chelating copper ions.

Since the adsorption results show that the type of alginate and the presence of histidine and beta amyloid can affect the removal of copper in aqueous media to varying degrees, it is important for future studies to conduct binding affinity analyses in order to present the intermolecular interactions that drive the copper-alginate-protein adsorption process and the structure-function relationships quantitatively. In addition to its relevance complementing the adsorption results presented in this paper, binding affinity analysis is also considered part of the therapeutic agent discovery process, helping to develop drugs that selectively and specifically bind their targets.

## 3. Materials and Methods

### 3.1. Materials

Alginate samples were provided by FMC Biopolymer (USA). AlgCol refers to alginate rich in mannuronic monomers (M), whereas AlgGel corresponds to alginate with more guluronic monomers (G). Table 4 presents the source and the monomer distribution for both alginate samples. Calcium chloride, hydrochloric acid, sodium hydroxide, and L-histidine monohydrochloride were purchased from Synth (Brazil). The trihydrate copper nitrate was obtained from Sigma (USA), and the βA is from the American Peptide Company (USA). All the solutions were prepared with ultrapure water from a Milli-Q^®^ water system.

### 3.2. Adsorbent Preparation

Alginate beads were prepared using a peristaltic pump to constantly drop 2.5% *w*/*v* alginate solution into a 4.0% *w*/*v* CaCl_2_ solution. After 24 h, alginate beads were removed from the CaCl_2_ solution and rinsed with double distilled water until the rinse water pH became neutral and then stored in neutral ultrapure water at 4 °C before use.

The adsorption properties of the βA peptide were assessed using a film prepared by casting. After peptide dilution in 1,1,1,3,3,3-hexafluoro-2-propanol (HFP) to 1 mmol L^−1^ of βA 42, 35 µL aliquots were collected and dried in the hood for 90 min at room temperature. A vacuum centrifuge was used to remove the remaining HFP traces, and the samples were stored at −80 °C before use [81].

### 3.3. Adsorbent Characterization

#### 3.3.1. Morphology

Alginate bead diameter was measured with the ImageJ open software using an image of five wet particles to determine particle diameter. Bead morphology was probed by SEM after sample lyophilization at 35 mbar and −45 °C (freeze-dry system, Labcomco, Brazil) and sputter-coating with an nm-sized gold layer (SC7620, Polaron, England). Measurements were carried out using a LEO440i electron microscope (LEO Electron Microscopy, England, UK) at an accelerating voltage of 10 kV, current of 50 pA, and magnification of 1000× to 3000×.

#### 3.3.2. Crystallinity

The predominance of the crystalline structure of the alginate particles was investigated by *X*-ray diffraction using a Philips Analytical *X*-ray diffractometer (X’Pert-MPD, Netherlands) with a Cu K radiation (λ = 1.5406 × 10^−10^ m) source. Samples were analyzed at 40 kV and 40 pA, with spectra collected with 2θ ranging from 5 to 90° (steps of 0.2°).

#### 3.3.3. Metal-Binding Functional Groups

Fourier-transformed infrared spectroscopy with attenuated reflectance element (FTIR-ATR) was used to investigate modifications in the functional groups in the alginate beads before and after the adsorption experiments. Measurements were conducted with the Nicolet 6700 spectrophotometer (Thermo Scientific, Waltham, MA, USA), ranging from 4000 to 675 cm^−1^.

#### 3.3.4. Adsorbent Chemical Composition

Using XPS, surface chemical composition and Cu (II)-alginate interaction were probed using XPS. Measurements were in AlgCol beads, determined with a PHI VersaProbe II *X*-ray photoelectron spectrophotometer using a monochromatic Al *X*-rays source (1486.6 eV, 50 W, 100 μm spot size). High-resolution spectra were collected for C 1s, O 1s, and Cu 2p on the surfaces of beads before and after metal ion adsorption, and their respective data were treated using CASA XPS software.

### 3.4. Adsorption Experiments

Copper adsorption experiments were carried out with Cu(NO_3_)_2_ solution at pH 5.0, considering both the copper ion speciation and the slightly acidic condition found in inflammation tissues affected by AD. All adsorption tests were performed in the static mode, i.e., with alginate adsorbate particles added to the Cu(II) solutions and kept at 150 rpm shaking at 25 °C. Four systems were used to investigate the effect of histidine and βA on Cu(II) adsorption performance of alginate beads: (1) Alginate beads were added to Cu(II) solutions without amino acid or peptide addition; (2) alginate beads were first added to Cu(II) solution, and after reaching the adsorption equilibrium, histidine or βA (both at 100 µM) was added to the system; (3) the Cu(II) solution was preliminarily treated with histidine (100 µmol L^−1^) for 2 h, followed by alginate addition; (4) alginate beads were treated with histidine (100 µmol L^−1^) for 24 h, followed by the filtration of the beads and Cu(II) adsorption experiments. Additionally, the adsorption properties of βA films were determined individually.

Adsorption kinetics experiments were conducted by adding 6.0 g of alginate beads (wet basis) to a 500 mL solution of copper ions (0.47 mmol L^−1^) and collecting sample aliquots (0.5 to 1.0 mL) to measure Cu(II) using an atomic absorption spectrophotometer analyzer (AA-7000, Shimadzu, Japan). Copper adsorption capacity (*Q*, mmol/g) was determined according to the following:

(5) $$Q=\left(C_{0}-C_{t}\right)\frac{V}{w}$$

where C_0_ [mmol L^−1^] is the initial copper concentration, C_t_ [mmol L^−1^] is the copper concentration at *t* [h], *V* is the solution volume [L], and *w* [g] is the initial adsorbate weight. Kinetic models (pseudo-first-order and pseudo-second-order) were fitted to the adsorption capacities over time using the least-square method in the Origin^®^ software.

Adsorption isotherm parameters were calculated for each of the four methods described above, using 0.3 g of alginate beads for Cu(II) adsorption in solutions with metal concentrations ranging from 5 to 300 mg/L. Solution aliquots were collected after each system reached equilibrium based on the adsorption kinetics experiment results. Alginate adsorption capacity (*Q*) in the equilibrium was also determined for each Cu(II) concentration (*C*) based on equation (Equation (5)). These results were fitted to adsorption isotherm models—namely, the Langmuir and Freundlich models—using the least-square method in the Origin^®^ software.

## 4. Conclusions

In this work, alginate samples with two M/G ratios were used to study the interaction of copper with biomolecules, such as the competition for Cu(II) adsorption in the presence of histidine. The alginate composition did not affect copper ion adsorption capacity (2.51 ± 0.08 mmol/g for AlgGel and 2.41 ± 0.07 mmol/g for AlgCol). Both samples follow the Langmuir model and pseudo-second-order adsorption kinetics, which is reflected in bead surfaces with adsorption sites of energetic similarities that undergo a chemisorption process during copper adsorption. The guluronic monomers promote interactions with copper that are more stable than those of mannuronic monomers in the presence of histidine due to the bidentate chelation of the metal ion with the former monomer. The order of the addition of histidine influences adsorption the most, resulting in a reduction of copper adsorption on the beads. Histidine performance in the adsorption test was similar to the βA, demonstrating that histidine is a simple, but representative model of βA for metal ion competition assays.

## Figures and Tables

**Table 1:** Element chemical composition of the alginate adsorbents before and after copper ion adsorption or histidine addition based on XPS. The pristine alginate pH values correspond to the alginate beads’ storage condition.

Bead Composition	Total Atomic Concentration				
	C 1s	O 1s	N 1s	Ca 2p	Cu 2p
AlgGel pH 7	60.34	32.95	3.03	3.68	-
AlgGel pH 5	60.42	36.98	-	2.06	-
AlgGel-Cu(II)	57.47	39.02	1.07	0.92	1.52
(Cu(II) + His) + AlgGel	58.38	40.22	0.32	0.15	0.92

**Table 2:** Adsorption kinetic parameters for the pseudo-first-order and pseudo-second-order kinetic models. Parameters were retrieved by fitting the data for copper ion adsorption in AlgCol and AlgGel systems.

Adsorption Systems	Pseudo-First-Order				Pseudo-Second-Order			
	$q_{e}$ (mmol/g)	$k_{1}$ (h^−1^)	*R* ^2^	*χ* ^2^	$q_{e}$ (mmol/g)	$k_{2}$ (h^−1/2^)	*R* ^2^	*χ* ^2^
AlgCol-Cu(II)	1.15 ± 0.02	0.70 ± 0.04	0.94	0.12	1.21 ± 0.03	0.92 ± 0.16	0.94	0.11
(Cu(II)-His)-AlgCol	1.19 ± 0.02	0.63 ± 0.05	0.96	0.08	1.26 ± 0.02	0.73 ± 0.08	0.98	0.05
(AlgCol-His)-Cu(II)	1.18 ± 0.01	0.80 ± 0.06	0.98	0.20	1.25 ± 0.01	1.06 ± 0.09	0.98	0.05
AlgGel-Cu(II)	0.73 ± 0.02	0.85 ± 0.12	0.88	0.17	0.78 ± 0.01	1.71 ± 0.21	0.97	0.04
(Cu(II)-His)-AlgGel	0.72 ± 0.03	0.43 ± 0.05	0.95	0.18	0.76 ± 0.03	0.91 ± 0.12	0.98	0.16
(AlgGel-His)-Cu(II)	0.72 ± 0.02	0.48 ± 0.07	0.90	0.07	0.77 ± 0.02	1.24 ± 0.36	0.90	0.02

**Table 3:** Isotherm parameters for the Langmuir and Freundlich models. Parameters were retrieved by inputting the data for copper ion adsorption in AlgCol and AlgGel systems.

Adsorption Systems	Langmuir				Freundlich			
	$q_{max}$ (mmol/g)	b (L/mmol)	*R* ^2^	*χ* ^2^	$q_{max}$ (mmol/g)	b (L/mmol)	*R* ^2^	*χ* ^2^
AlgCol-Cu(II)	2.41 ± 0.07	8.40 ± 0.95	0.99	0.09	1.91 ± 0.11	3.53 ± 0.53	0.89	0.65
(Cu(II)-His)-AlgCol	2.34 ± 0.16	11.6 ± 4.01	0.82	0.79	1.89 ± 0.14	3.39 ± 0.67	0.85	8.88
(AlgCol-His)-Cu(II)	2.38 ± 0.21	4.44 ± 1.49	0.94	0.35	1.83 ± 0.09	2.36 ± 0.28	0.95	1.71
(Cu(II)-AlgCol)-His	1.37 ± 0.03	13.1 ± 1.26	0.99	0.06	1.19 ± 0.08	3.66 ± 0.72	0.83	1.89
AlgGel-Cu(II)	2.51 ± 0.08	9.74 ± 1.34	0.98	0.36	2.02 ± 0.11	4.24 ± 0.72	0.87	0.57
(Cu(II)-His)-AlgGel	2.02 ± 0.16	10.8 ± 3.49	0.87	0.70	1.64 ± 0.16	3.80 ± 1.04	0.71	5.65
(AlgGel-His)-Cu(II)	2.04 ± 0.16	4.67 ± 1.52	0.93	0.77	1.50 ± 0.07	2.97 ± 0.39	0.93	1.05
(Cu(II)-AlgGel)-His	2.05 ± 0.14	4.61 ± 1.35	0.93	0.36	1.44 ± 0.09	2.93 ± 0.46	0.90	3.37
AlgGel-Cu(II)	2.56 ± 0.08	6.48 ± 1.15	0.95	-	2.09 ± 0.08	5.62 ± 1.31	0.81	-
(Cu(II)-BA)-AlgGel	1.96 ± 0.02	19.3 ± 2.97	0.92	-	1.81 ± 0.03	16.3 ± 4.65	0.72	-

**Table 4:** The source, molar fraction (F) of individual monomers, and the respective blocks of mannuronic (M) and guluronic (G) monomers on AlgGel and AlgCol [80].

Sample	Source	M/G	F_M_	F_G_	F_MM_	F_GG_
AlgCol	*Macrocystis pyrifera*	1.94	0.66	0.34	0.49	0.17
AlgGel	*Lamninaria hyperborea*	0.61	0.38	0.62	0.18	0.42

## Data Availability

Not applicable.

## Supplementary Materials

The following supporting information can be downloaded at: https://www.mdpi.com/article/10.3390/molecules27238334/s1, Figure S1: Image of alginate beads for copper ion adsorption. (A) AlgGel, (B) AlgGel+Cu(II), (C) AlgCol, and (D) AlgCol + Cu (II). The ruler scale is on mm; Figure S2: High-resolution Cu2p spectra for AlgGEL after copper adsorption and histidine addition; Figure S3: FTIR spectra for (A) AlgCol and (B) AlgGel before and after copper adsorption in the presence of histidine; Figure S4: FTIR spectra for AlgCol and AlgGel sodium salt; Table S1: Mean diameter for alginate beads. Results were obtained using the ImageJ® software. Standard deviation represents the average of three measurements; Table S2: Intervals of COO^−^ symmetric and asymmetric vibration bands (Δν) on the FTIR spectra of the studied alginate systems. Reference citation [58,59,60,61,62].

## Sample Availability

Please contact the authors if additional information on sample preparation is necessary.

## References

[1] 2022 Alzheimer’s Disease Facts and Figures. https://www.alz.org/media/Documents/alzheimers-facts-and-figures.pdf.

[2] Braak H., Braak E. (1991). Neuropathological Stageing of Alzheimer-Related Changes. Acta Neuropathol..

[3] Hardy J.A., Higgins G.A. (1992). Alzheimer’s Disease: The Amyloid Cascade Hypothesis. Science.

[4] Gaggelli E., Kozlowski H., Valensin D., Valensin G. (2006). Copper Homeostasis and Neurodegenerative Disorders (Alzheimer’s, Prion, and Parkinson’s Diseases and Amyotrophic Lateral Sclerosis). Chem. Rev..

[5] Hampel H., Hardy J., Blennow K., Chen C., Perry G., Kim S.H., Villemagne V.L., Aisen P., Vendruscolo M., Iwatsubo T. (2021). The Amyloid-β Pathway in Alzheimer’s Disease. Mol. Psychiatry.

[6] Atwood C.S., Obrenovich M.E., Liu T., Chan H., Perry G., Smith M.A., Martins R.N. (2003). Amyloid-Beta: A Chameleon Walking in Two Worlds: A Review of the Trophic and Toxic Properties of Amyloid-Beta. Brain Res. Rev..

[7] Vetrivel K.S., Thinakaran G. (2006). Amyloidogenic Processing of Beta-Amyloid Precursor Protein in Intracellular Compartments. Neurology.

[8] Opazo C., Huang X., Cherny R.A., Moir R.D., Roher A.E., White A.R., Cappai R., Masters C.L., Tanzi R.E., Inestrosa N.C. (2002). Metalloenzyme-like Activity of Alzheimer’s Disease Beta-Amyloid. Cu-Dependent Catalytic Conversion of Dopamine, Cholesterol, and Biological Reducing Agents to Neurotoxic H(2)O(2). J. Biol. Chem..

[9] Raffa D.F., Rickard G.A., Rauk A. (2007). Ab Initio Modelling of the Structure and Redox Behaviour of Copper(I) Bound to a His-His Model Peptide: Relevance to the Beta-Amyloid Peptide of Alzheimer’s Disease. J. Biol. Inorg. Chem..

[10] Zhou Z., Chen S., Huang Y., Gu B., Li J., Wu C., Yin P., Zhang Y., Li H. (2022). Simultaneous Visualization and Quantification of Copper (II) Ions in Alzheimer’s Disease by a near-Infrared Fluorescence Probe. Biosens. Bioelectron..

[11] Linder M.C., Hazegh-Azam M. (1996). Copper Biochemistry and Molecular Biology. Am. J. Clin. Nutr..

[12] Huang X., Cuajungco M.P., Atwood C.S., Hartshorn M.A., Tyndall J.D., Hanson G.R., Stokes K.C., Leopold M., Multhaup G., Goldstein L.E. (1999). Cu (II) Potentiation of Alzheimer Abeta Neurotoxicity. Correlation with Cell-Free Hydrogen Peroxide Production and Metal Reduction. J. Biol. Chem..

[13] Yugay D., Goronzy D.P., Kawakami L.M., Claridge S.A., Song T.-B., Yan Z., Xie Y.-H., Gilles J., Yang Y., Weiss P.S. (2016). Copper Ion Binding Site in β-Amyloid Peptide. Nano Lett..

[14] Streltsov V.A., Titmuss S.J., Epa V.C., Barnham K.J., Masters C.L., Varghese J.N. (2008). The Structure of the Amyloid-Beta Peptide High-Affinity Copper II Binding Site in Alzheimer Disease. Biophys. J..

[15] Cherny R.A., Atwood C.S., Xilinas M.E., Gray D.N., Jones W.D., McLean C.A., Barnham K.J., Volitakis I., Fraser F.W., Kim Y. (2001). Treatment with a Copper-Zinc Chelator Markedly and Rapidly Inhibits Beta-Amyloid Accumulation in Alzheimer’s Disease Transgenic Mice. Neuron.

[16] Fasae K.D., Abolaji A.O., Faloye T.R., Odunsi A.Y., Oyetayo B.O., Enya J.I., Rotimi J.A., Akinyemi R.O., Whitworth A.J., Aschner M. (2021). Metallobiology and Therapeutic Chelation of Biometals (Copper, Zinc and Iron) in Alzheimer’s Disease: Limitations, and Current and Future Perspectives. J. Trace Elem. Med. Biol..

[17] Zheng H., Fridkin M., Youdim M.B.H. (2010). Site-Activated Chelators Derived from Anti-Parkinson Drug Rasagiline as a Potential Safer and More Effective Approach to the Treatment of Alzheimer’s Disease. Neurochem. Res..

[18] Onsøyen E., Imeson A.P. (1997). Alginates. Thickening and Gelling Agents for Food.

[19] Draget K.I., Skjåk-Braek G., Smidsrød O. (1997). Alginate Based New Materials. Int. J. Biol. Macromol.

[20] Haug A., Larsen B., Smidsrød O. (1974). Uronic Acid Sequence in Alginate from Different Sources. Carbohydr. Res..

[21] Abka-khajouei R., Tounsi L., Shahabi N., Patel A.K., Abdelkafi S., Michaud P. (2022). Structures, Properties and Applications of Alginates. Mar. Drugs.

[22] Puscaselu R.G., Lobiuc A., Dimian M., Covasa M. (2020). Alginate: From Food Industry to Biomedical Applications and Management of Metabolic Disorders. Polymers.

[23] Gao C., Tang F., Gong G., Zhang J., Hoi M.P.M., Lee S.M.Y., Wang R. (2017). PH-Responsive Prodrug Nanoparticles Based on a Sodium Alginate Derivative for Selective Co-Release of Doxorubicin and Curcumin into Tumor Cells. Nanoscale.

[24] Hariyadi D.M., Islam N. (2020). Current Status of Alginate in Drug Delivery. Adv. Pharmacol. Pharm. Sci..

[25] Barbu A., Neamtu B., Zăhan M., Iancu G.M., Bacila C., Mireșan V. (2021). Current Trends in Advanced Alginate-Based Wound Dressings for Chronic Wounds. J. Pers. Med..

[26] Tao B., Deng Y., Song L., Ma W., Qian Y., Lin C., Yuan Z., Lu L., Chen M., Yang X. (2019). BMP2-Loaded Titania Nanotubes Coating with PH-Responsive Multilayers for Bacterial Infections Inhibition and Osteogenic Activity Improvement. Colloids. Surf. B Biointerfaces.

[27] Ikeda A., Takemura A., Ono H. (2000). Preparation of Low-Molecular Weight Alginic Acid by Acid Hydrolysis. Carbohydr. Polym..

[28] Davis T.A., Volesky B., Mucci A. (2003). A Review of the Biochemistry of Heavy Metal Biosorption by Brown Algae. Water Res..

[29] Braccini I., Pérez S. (2001). Molecular Basis of C (2+)-Induced Gelation in Alginates and Pectins: The Egg-Box Model Revisited. Biomacromolecules.

[30] Papageorgiou S.K., Katsaros F.K., Kouvelos E.P., Nolan J.W., Le Deit H., Kanellopoulos N.K. (2006). Heavy Metal Sorption by Calcium Alginate Beads from Laminaria Digitata. J. Hazard Mater..

[31] Aneem T.H., Wong S.Y., Afrin H., Nurunnabi M., Li X., Arafat M.T. (2021). Investigation of Coagulation Process of Wet-Spun Sodium Alginate Polymannuronate Fibers with Varied Functionality Using Organic Coagulants and Cross-Linkers. Mater. Today Chem..

[32] Xiao C., Liu H., Lu Y., Zhang L. (2001). Blend Films from Sodium Alginate and Gelatin Solutions. J. Macromol. Sci. Part A.

[33] Chapman V.J., Chapman D.J. (1980). Seaweeds and Their Uses.

[34] Gu C., Sun B., Wu W., Wang F., Zhu M. (2007). Synthesis, Characterization of Copper-Loaded Carboxymethyl-Chitosan Nanoparticles with Effective Antibacterial Activity. Macromol. Symp..

[35] Davis T.A., Llanes F., Volesky B., Mucci A. (2003). Metal Selectivity of Sargassum Spp. and Their Alginates in Relation to Their α-l-Guluronic Acid Content and Conformation. Environ. Sci. Technol..

[36] Anuradha G. (2016). V Studies on Structural, Optical, Mechanical Properties of Undoped and Doped l-Histidine Monohydrate Monohydrochloride (LHMHCL). Optik.

[37] Gómez-Ordóñez E., Rupérez P. (2011). FTIR-ATR Spectroscopy as a Tool for Polysaccharide Identification in Edible Brown and Red Seaweeds. Food Hydrocoll..

[38] Lawrie G., Keen I., Drew B., Chandler-Temple A., Rintoul L., Fredericks P., Grøndahl L. (2007). Interactions between Alginate and Chitosan Biopolymers Characterized Using FTIR and XPS. Biomacromolecules.

[39] Papageorgiou S.K., Kouvelos E.P., Favvas E.P., Sapalidis A.A., Romanos G.E., Katsaros F.K. (2010). Metal-Carboxylate Interactions in Metal–Alginate Complexes Studied with FTIR Spectroscopy. Carbohydr. Res..

[40] Sakugawa K., Ikeda A., Takemura A., Ono H. (2004). Simplified Method for Estimation of Composition of Alginates by FTIR. J. Appl. Polym. Sci..

[41] Trandafilović L.V., Whiffen R.K., Dimitrijević-Branković S., Stoiljković M., Luyt A.S., Djoković V. (2014). ZnO/Ag Hybrid Nanocubes in Alginate Biopolymer: Synthesis and Properties. Chem. Eng. J..

[42] di Cocco M.E., Bianchetti C., Chiellini F. (2003). 1H NMR Studies of Alginate Interactions with Amino Acids. J. Bioact. Compat. Polym..

[43] Sartori C., Finch D.S., Ralph B., Gilding K. (1997). Determination of the Cation Content of Alginate Thin Films by FTi.r. Spectroscopy. Polymer.

[44] van Hoogmoed C.G., Busscher H.J., de Vos P. (2003). Fourier Transform Infrared Spectroscopy Studies of Alginate-PLL Capsules with Varying Compositions. J. Biomed. Mater. Res. A.

[45] Bertagnolli C. (2013). Bioadsorção de Cromo Na Alga Sargassum Filipendula e Em Seus Derivados. Ph.D. Thesis.

[46] Mehrotra R.C., Bohra R. (1983). Metal Carboxylates.

[47] Alcock N.W., Tracy V.M., Waddington T.C. (1976). Acetates and Acetato-Complexes. Part 2. Spectroscopic Studies. J. Chem. Soc. Dalton. Trans..

[48] Tackett J.E. (1989). FT-IR Characterization of Metal Acetates in Aqueous Solution. Appl. Spectrosc..

[49] Nakamoto K. (2008). Infrared and Raman Spectra of Inorganic and Coordination Compounds.

[50] Deacon G.B., Phillips R.J. (1980). Relationships between the Carbon-Oxygen Stretching Frequencies of Carboxylato Complexes and the Type of Carboxylate Coordination. Coord. Chem. Rev..

[51] Bertagnolli C., Uhart A., Dupin J.-C., da Silva M.G.C., Guibal E., Desbrieres J. (2014). Biosorption of Chromium by Alginate Extraction Products from Sargassum Filipendula: Investigation of Adsorption Mechanisms Using *X*-ray Photoelectron Spectroscopy Analysis. Bioresour. Technol..

[52] Oliveira R.C., Hammer P., Guibal E., Taulemesse J.-M., Garcia O. (2014). Characterization of Metal–Biomass Interactions in the Lanthanum (III) Biosorption on Sargassum Sp. Using SEM/EDX, FTIR, and XPS: Preliminary Studies. Chem. Eng. J..

[53] Jodra Y., Mijangos F. (2001). Ion Exchange Selectivities of Calcium Alginate Gels for Heavy Metals. Water Sci. Technol..

[54] Khotimchenko M., Kovalev V., Khotimchenko Y. (2008). Comparative Equilibrium Studies of Sorption of Pb (II) Ions by Sodium and Calcium Alginate. J. Environ. Sci..

[55] Kleinübing S.J., Vieira R.S., Beppu M.M., Guibal E., Silva M.G.C. (2010). da Characterization and Evaluation of Copper and Nickel Biosorption on Acidic Algae Sargassum Filipendula. Mater. Res..

[56] Moulder J.F., Chastain J. (1992). Handbook of *X*-ray Photoelectron Spectroscopy: A Reference Book of Standard Spectra for Identification and Interpretation of XPS Data.

[57] Chen J.P., Hong L., Wu S., Wang L. (2002). Elucidation of Interactions between Metal Ions and Ca Alginate-Based Ion-Exchange Resin by Spectroscopic Analysis and Modeling Simulation. Langmuir.

[58] Biesinger M.C., Lau L.W.M., Gerson A.R., Smart R.S.C. (2010). Resolving Surface Chemical States in XPS Analysis of First Row Transition Metals, Oxides and Hydroxides: Sc, Ti, V, Cu and Zn. Appl. Surf. Sci..

[59] Roy A., Mukhopadhyay A.K., Das S.C., Bhattacharjee G., Majumdar A., Hippler R. (2019). Surface Stoichiometry and Optical Properties of Cux–TiyCz Thin Films Deposited by Magnetron Sputtering. Coatings.

[60] Wang Y., Lü Y., Zhan W., Xie Z., Kuang Q., Zheng L. (2015). Synthesis of Porous Cu 2 O/CuO Cages Using Cu-Based Metal–Organic Frameworks as Templates and Their Gas-Sensing Properties. J. Mater. Chem. A Mater..

[61] Beamson G., Briggs D. (1993). High Resolution XPS of Organic Polymers: The Scienta ESCA300 Database. J. Chem. Educ..

[62] Watts J.F., Wolstenholme J. (2019). The Electron Spectrum. An Introduction to Surface Analysis by XPS and AES.

[63] Chen J., Tendeyong F., Yiacoumi S. (1997). Equilibrium and Kinetic Studies of Copper Ion Uptake by Calcium Alginate. Environ. Sci. Technol..

[64] Kleinübing S.J., Gaia F., Bertagnolli C., Da Silva M.G.C. (2013). Extraction of Alginate Biopolymer Present in Marine Alga Sargassum Filipendula and Bioadsorption of Metallic Ions. Mater. Res..

[65] Guibal E. (2004). Interactions of Metal Ions with Chitosan-Based Sorbents: A Review. Sep. Purif. Technol..

[66] Lagergren S. (1907). Zur Theorie Der Sogenannten Adsorption Gelöster Stoffe. Z. Für Chem. Ind. Kolloide.

[67] Ho Y.S., McKay G. (2000). The Kinetics of Sorption of Divalent Metal Ions onto Sphagnum Moss Peat. Water Res..

[68] Chiou M.S., Li H.Y. (2003). Adsorption Behavior of Reactive Dye in Aqueous Solution on Chemical Cross-Linked Chitosan Beads. Chemosphere.

[69] Nai-yu Z., Yan-xia Z., Xiao F., Li-jun H. (1994). Effects of Composition and Structure of Alginates on Adsorption of Divalent Metals. Chin. J. Oceanol. Limnol..

[70] Cozzi D., Desideri P.G., Lepri L. (1969). The Mechanism of Ion Exchange with Alginic Acid. J. Chromatogr. A.

[71] Taketa T.B., Mahl C.R.A., Calais G.B., Beppu M.M. (2021). Amino Acid-Functionalized Chitosan Beads for in Vitro Copper Ions Uptake in the Presence of Histidine. Int. J. Biol. Macromol..

[72] Jaiana Kleinübing S. (2009). Bioadsorção Competitiva Dos Ions Niquel e Cobre Em Alginato e Alga Marinha Sargassum Filipendula.

[73] Awala H.A., El Jamal M.M. (2011). Equilibrium and Kinetics Study of Adsorption of Some Dyes onto Feldspar. J. Univ. Chem. Technol. Metall..

[74] Kumar P.S., Kirthika K. (2009). Equilibrium and Kinetic Study of Adsorption of Nickel from Aqueous Solution onto Bael Tree Leaf Powder. J. Eng. Sci. Technol..

[75] Ngah W.S.W., Fatinathan S. (2008). Adsorption of Cu (II) Ions in Aqueous Solution Using Chitosan Beads, Chitosan–GLA Beads and Chitosan–Alginate Beads. Chem. Eng. J..

[76] Smidsrød O. (1974). Molecular Basis for Some Physical Properties of Alginates in the Gel State. Faraday Discuss. Chem. Soc..

[77] Vieira R.S., Beppu M.M. (2005). Mercury Ion Recovery Using Natural and Crosslinked Chitosan Membranes. Adsorption.

[78] Eftekharzadeh B., Khodagholi F., Abdi A., Maghsoudi N. (2010). Alginate Protects NT2 Neurons against H2O2-Induced Neurotoxicity. Carbohydr. Polym..

[79] Zhou R., Shi X.-Y., Bi D.-C., Fang W.-S., Wei G.-B., Xu X. (2015). Alginate-Derived Oligosaccharide Inhibits Neuroinflammation and Promotes Microglial Phagocytosis of β-Amyloid. Mar. Drugs.

[80] Vaz J.M. (2012). Preparação e Caracterização de Biofilmes Ativos à Base de Alginato de Diferentes Estruturas Poliméricas Reticuladas Com Cálcio. Masters Thesis.

[81] Stine W.B., Dahlgren K.N., Krafft G.A., LaDu M.J. (2003). In Vitro Characterization of Conditions for Amyloid-β Peptide Oligomerization and Fibrillogenesis. J. Biol. Chem..

