# Supplementary material for: Role of Alginate Composition on Copper Ion Uptake in the Presence of Histidine or Beta-Amyloid
Source: Molecules. 2022 Nov 29;27(23):8334. doi: 10.3390/molecules27238334 (PMC9735935; doi:10.3390/molecules27238334)
Supplement: Supplementary file 1 [file molecules-27-08334-s001.zip › molecules-2010022-supplementary.pdf]

molecules

SUPPORTING INFORMATION

Role of Alginate Composition on Copper Ion Uptake in the Presence of Histidine or Beta-Amyloid

Cynthia Regina Albrecht Mahl, Rogério Aparecido Bataglioli, Guilherme Bedeschi Calais, Thiago Bezerra Taketa and Marisa Masumi Beppu *

School of Chemical Engineering, University of Campinas, UNICAMP, 500, Albert Einstein Av., Campinas 13083-852, SP, Brazil; cramahleq@gmail.com (C.R.A.M.); rbatagl@unicamp.br (R.A.B.);
g262773@dac.unicamp.br (G.B.C.); thiagobt@gmail.com (T.B.T.)
* Correspondence: beppu@unicamp.br; Tel.: +55-19-3521-3882

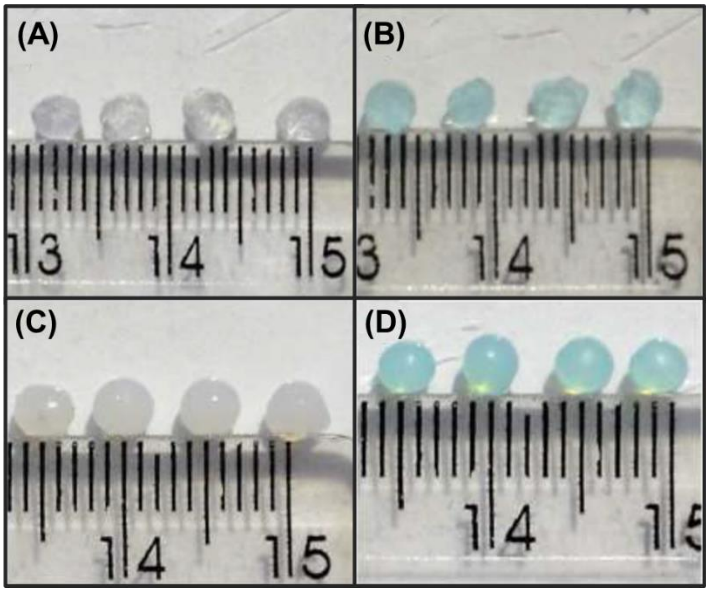

Figure S1. Image of alginate beads for copper ion adsorption. (A) AlgGel, (B) AlgGel+Cu(II), (C) AlgCol, and (D) AlgCol+Cu (II). The ruler scale is on mm.

Table S1. Mean diameter for alginate beads. Results were obtained using the ImageJ® software. Standard deviation represents the average of three measurements.

Bead components	Mean Diameter (mm)
AlgGel	2.88 ± 025
AlgGel+Cu(II)	2.95 ± 0.36
(Cu(II)+His)+ AlgGel	2.96 ± 0.18
AlgCol	3.01 ± 0.11
AlgCol+Cu(II)	2.87 ± 0.33
(Cu(II)+His)+ AlgGel	2.90 ± 0.32

Table S2. Intervals of COO^- symmetric and asymmetric vibration bands ($\Delta\nu$) on the FTIR spectra of the studied alginate systems.

Bead composition	$COO^-_{asymmetric}$ (cm^{-1})	$COO^-_{symmetric}$ (cm^{-1})	$\Delta\nu$ (cm^{-1})
AlgGel sodium	1615	1419	196
AlgGel calcium	1600	1418	182
AlgGel+Cu(II)	1600	1415	185
(Cu(II)+His)+AlgGel	1600	1411	189
AlgCol sodium	1613	1415	198
AlgCol calcium	1600	1412	188
AlgCol+Cu(II)	1600	1414	186
(Cu(II)+His)+AlgCol	1597	1417	180

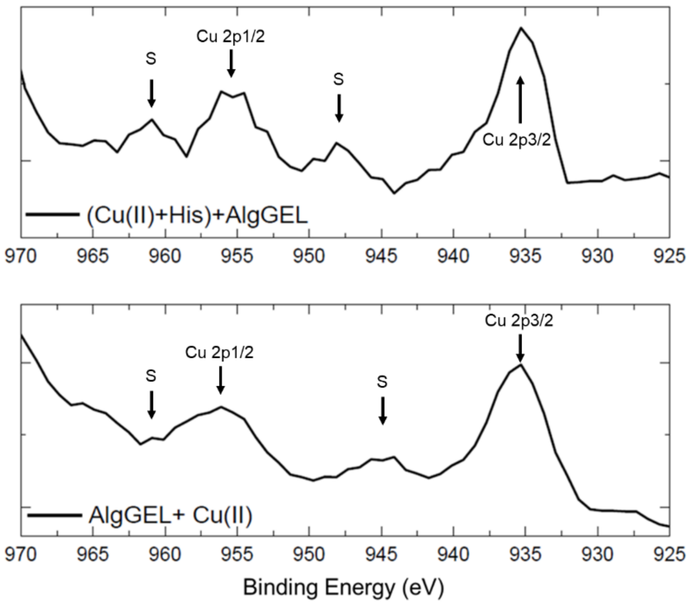

Figure S2. High-resolution Cu2p spectra for AlgGEL after copper adsorption and histidine addition.

The extensive noise signal in Cu 2p spectra is observed due to the short exposure time of the sample to radiation, which was required to avoid copper photoreduction by the action of X-rays during spectrum acquisition. The peaks at 2p3/2 = 935.2 and 2p1/2 = 955.2 eV indicate the presence of the Cu(II) on the surface of the samples [58–62], which is also reinforced by the presence of shake-up lines (S) at 945 and 965 eV for Cu 2p3/2 and 2p1/2. This occurs when an atom's innermost electrons are stripped away. As a result, its valence electrons undergo a reorganization, leading to a rise of the electron to a higher energy level (shake-up level). The secondary peak is formed due to energy loss in this process. These peaks are normally observed in transitions of some metallic compounds that have 3d or 4f unpaired electrons, such as copper ions [62]. The shake-up lines are an indication of an open $3d^9$ Cu(II) sphere, indicating the presence of CuO on the surface of the sphere. The same is observed when histidine is added to the system.

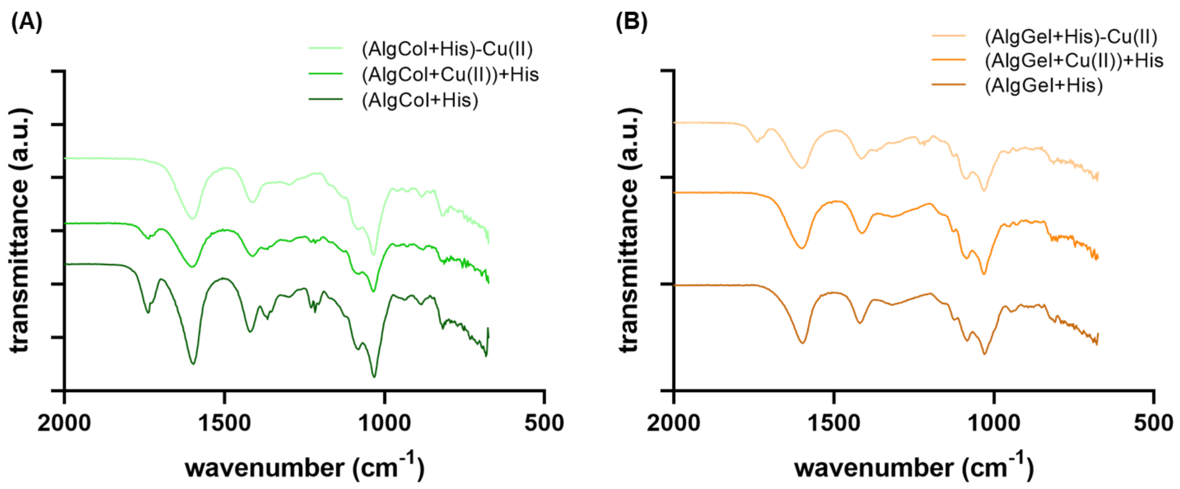

Figure S3. FTIR spectra for (A) AlgCol and (B) AlgGel during copper adsorption in the presence of histidine.

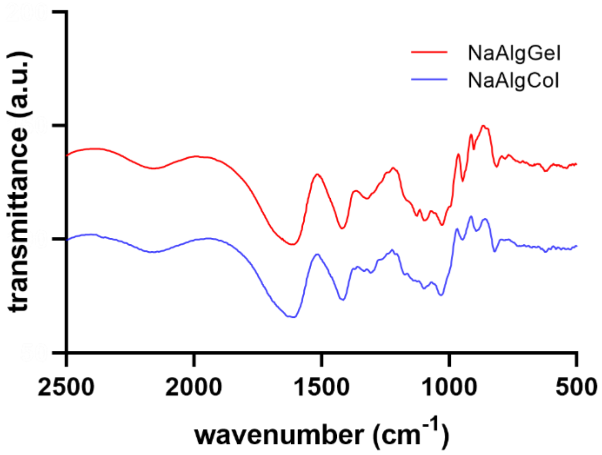

Figure S4. FTIR spectra for AlgCol and AlgGel sodium salt.
